# Morphological Spectrum of Orbitoocular Diseases in a Tertiary Health Centre in Keffi, North Central Nigeria

**DOI:** 10.1155/2015/619414

**Published:** 2015-10-20

**Authors:** Ifeyinwa Mary Onwubuya, Tunde Mark Owoyele, Olaejirinde Olaniyi Olaofe, Kevin Nwabueze Ezike

**Affiliations:** ^1^Department of Morbid Anatomy and Histopathology, Federal Medical Centre, Keffi 770108, Nigeria; ^2^Department of Ophthalmology, Federal Medical Centre, Keffi 770108, Nigeria; ^3^Department of Morbid Anatomy & Forensic Medicine, Obafemi Awolowo University, Ile-Ife 220282, Nigeria; ^4^Pathology Unit, Asokoro District Hospital, Abuja 900231, Nigeria

## Abstract

*Aim*. The aim of this study was to carry out a retrospective clinicopathological analysis of the ocular lesions requiring biopsy seen in the Department of Histopathology, Federal Medical Centre (FMC), Keffi, in North Central Nigeria. *Materials and Method*. A retrospective review of the clinicopathologic profile of orbitoocular lesions diagnosed at the FMC, Keffi, was done. Clinical and pathological data were obtained from the patients' clinical records and original biopsy reports, respectively. *Results*. Sixty-six cases of orbitoocular lesions were reviewed for this study. Of the 54 cases investigated, 28 were HIV negative while 26 were HIV positive (37.1% of all cases). There were 30 cases of Ocular Surface Squamous Neoplasia (OSSN) with a male-to-female ratio of 0.9 : 1. Squamous cell carcinoma (SCC) was the most frequent OSSN with 17 cases. The mean age of cases of SCC is 37.1 ± 7.6 SD (years). The mean age of carcinoma in situ is 35.8 ± 11.4 years. *Conclusion*. There was no significant difference in the sex distribution of patients with OSSN. It is probable that a diagnosis of squamous cell carcinoma may be encountered in about a year after diagnosis of a carcinoma in situ especially if the in situ carcinoma is left untreated or improperly treated.

## 1. Introduction

Orbitoocular diseases are an important cause of morbidity and mortality in our population and these lesions constitute a large proportion of biopsies received in the histopathology department of our hospital. Some of the lesions commonly encountered among others include biopsy specimen for papillomas, degenerative changes such as pterygium and pinguecula, and enucleation specimen for malignant tumours such as retinoblastoma and squamous cell carcinoma.

Local and international variations in the pattern of orbitoocular diseases are noted especially with regard to age, sex, site, and possible aetiological factors. A 15-year histopathological review of orbitoocular diseases in UBTH, Benin City, by Aligbe et al. [1] revealed that 43% of all the diseases were found in children under 15 years old and 41.9% of the lesions were malignant. The conjunctiva was also found to be the commonest site for the lesions and the commonest lesion in this site was squamous cell carcinoma [1]. A similar study in Lagos by Anunobi et al. [2] revealed that males presented more frequently than females with these lesions, with 51.1% of cases occurring in children 15 years old and below. This study also revealed that squamous cell carcinoma was the commonest of all malignant conjunctival lesions accounting for four out of five cases of malignant conjunctival lesions while retinoblastoma was the most common orbitoocular malignancy making up 85% of all such malignancies, with a peak age of presentation of 1–5 years. Inflammatory lesions such as panophthalmitis were also noted as common indications for enucleation in this study [2]. The study by Bekibele and Oluwasola [3] in Ibadan revealed that squamous cell carcinoma of the conjunctiva accounting for 12.1% of all malignancies was the commonest malignancy of the adult age group. They however did not find any cases of malignant melanoma or Kaposi sarcoma in the study. A 21-year review of 244 orbital tumours in Japan by Ohtsuka et al. [4] showed that 89% of all the cases were primary orbital tumours while secondary tumours arising from contiguous spaces accounted for 9% of all the cases, and 2% were metastatic tumours. The most common tumour found in the 0–9-year age group was dermoid cyst, accounting for 26% of all cases, followed by optic nerve glioma (11%). In patients above 40 years old, malignant lymphoma was the commonest tumour seen accounting for 31% of cases.

Some ocular disorders are known to occur at all stages of HIV infection. Squamous cell carcinoma of the orbit has been linked with infection by HIV I and HIV II in some studies. Osahon and Onunu [5] in their 5-year prospective study, “Ocular Disorders in Patients with HIV Infection in UBTH Benin” revealed that 21 of 526 patients who were HIV positive had ocular diseases, giving a prevalence rate of 4.0%. The lesions seen in these patients were herpes zoster ophthalmicus, squamous cell carcinoma, Kaposi sarcoma, and CMV retinitis. Bekibele and Oluwasola [3] recorded that only 2 out of the 8 patients in their study who had squamous cell carcinoma tested positive for HIV and therefore could not further evaluate any link between the infection and the malignancy.

Pathological profiles of orbitoocular lesions when characterised according to demographic data and clinical findings provide information on the existence and prevalence of these diseases and may help guide diagnosis prior to biopsy or resection and for determination of treatment strategy.

There is however paucity of studies on the pattern and characteristics of these diseases in this region (North Central Nigeria), thus limiting available information applicable to the area.

Therefore, this study seeks to review and update data for demography (age and sex) and prevalence of common orbitoocular lesions in Keffi, as well as describe their clinicopathological features, in comparison to other parts of the country and the world.

## 2. Materials and Methods

This was a retrospective review of all orbitoocular lesions diagnosed at the histopathology department of the FMC, Keffi, a tertiary health care facility in north central zone of Nigeria.

Patients' demographic data and pathological diagnosis were retrieved from original biopsy reports while clinical data were obtained from the patients' clinical records. The patients' age, sex, clinical features, indications for ocular biopsies, and pathological diagnosis were noted.

The original slides were retrieved and reviewed, and, where necessary, fresh sections were cut from archival tissue paraffin blocks and stained by routine haematoxylin and eosin (H&E).

The data obtained were analyzed for differences in proportion using chi square by the Statistical Package for Social Sciences (SPSS) version 16.0. (*p* is significant at <0.05.)

Ethical clearance for the study was obtained from the ethics and research committee of the Federal Medical Centre, Keffi, Nasarawa State, Nigeria.

## 3. Results

A total of sixty-six cases of orbitoocular lesions were seen within the study period. There were 40 males and 26 females with an overall male-to-female ratio of 1.5 : 1.

**Figure 1:**
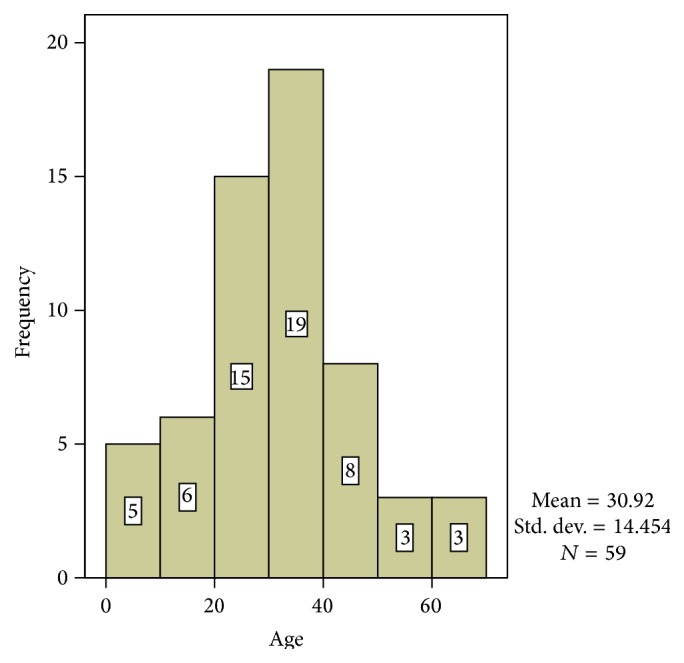
Age distribution of ocular lesions.

The overall mean age was 30.9 ± 14.5 SD (years) while the age range was 2–70 years. The highest frequency of cases was seen in the 30–39 years' age group with 19 cases while there were 15 cases in the 20–29 years' age group. Other age groups and their frequencies are shown in Figure 1. The mean age of cases of squamous cell carcinoma (SCC) is 37.1 ± 7.6 SD (years). The age range for SCC is 25–53 years. The mean age of carcinoma in situ is 35.8 ± 11.4 SD years. The age range was 24–60 years.

**Figure 2:**
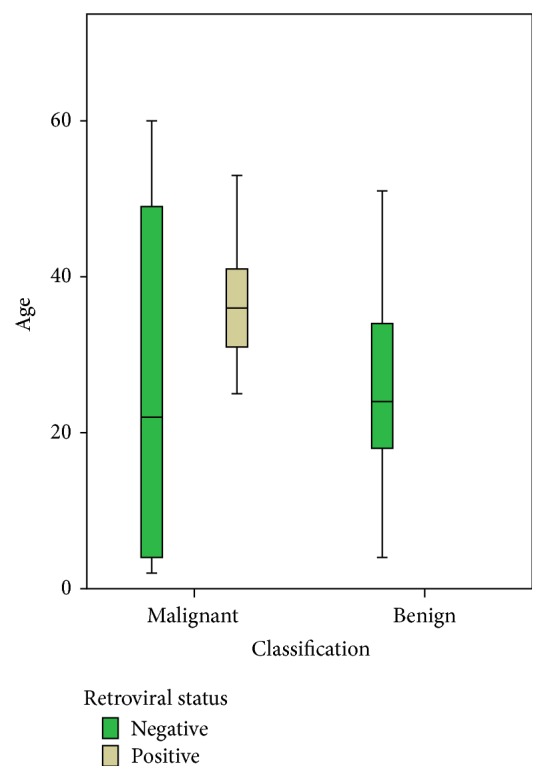
Box and plot chart of mean age and age variation of HIV positive and negative cases in relation to malignant status of the ocular lesions.

HIV status was ascertained in only 54 cases. Of these, 26 cases were HIV positive while 28 were HIV negative.  Figure 2 shows the age variations in HIV positive and negative cases of both benign and malignant ocular lesions.

**Figure 3:**
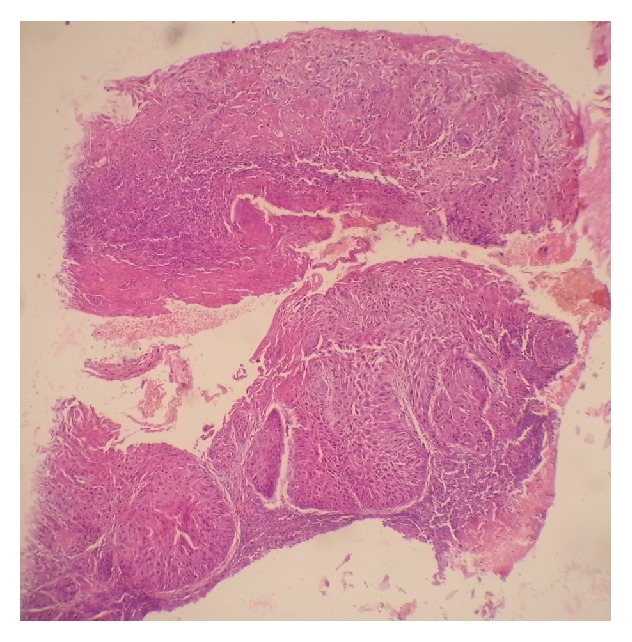
Photomicrograph of an invasive squamous carcinoma of the conjunctiva.

**Figure 4:**
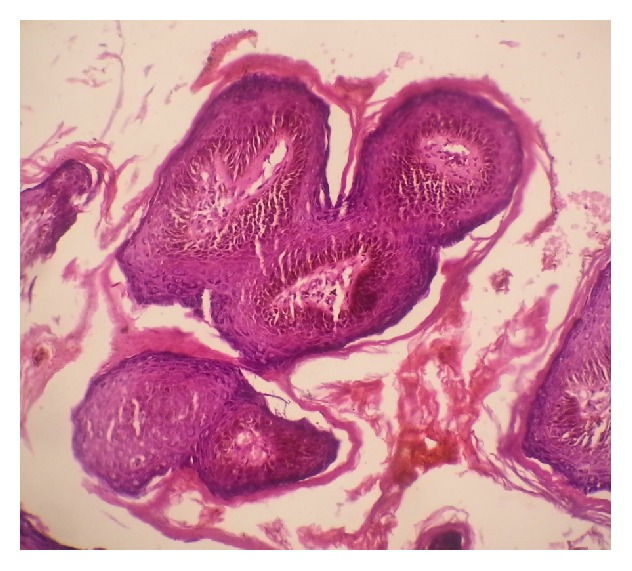
Photomicrograph of a conjunctival squamous papilloma.

**Figure 5:**
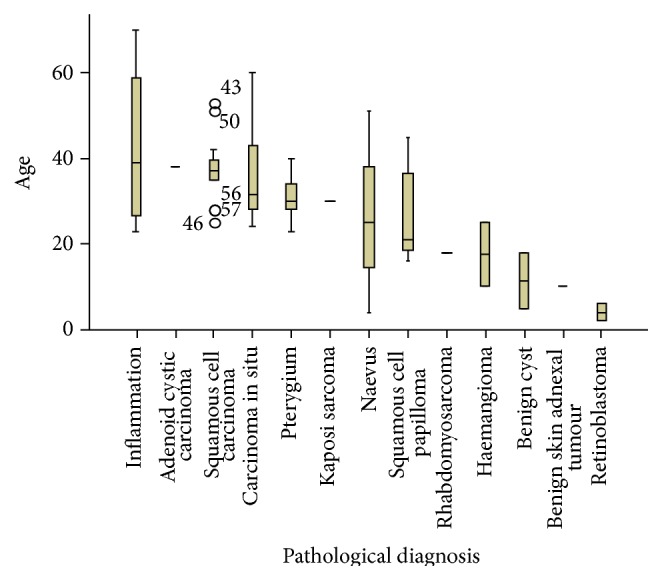
Box plot chart of mean age and age variation of the various histologic diagnoses.

There were 30 cases of Ocular Surface Squamous Neoplasia (OSSN) with males accounting for 14 cases (46.7%) and females accounting for 16 cases (53.3%) (male-to-female ratio of 1.1 : 1). Squamous cell carcinoma was the most frequent lesion and was found in 17 cases (24.3%).  Figure 3 shows a photomicrograph of an invasive squamous carcinoma of the conjunctiva in a 35-year-old HIV positive man. Carcinoma in situ was found in 13 cases (18.6%). Squamous cell papilloma was found in 9 cases (12.9%).  Figure 4 shows a photomicrograph of a conjunctival squamous papilloma in a 43-year-old man who was HIV negative. Pterygium was found in 6 cases (8.6%). Four cases were inflammatory lesions. Retinoblastoma, haemangioma, and benign cyst accounted for 2 cases each. Adenoid cystic carcinoma, benign skin adnexal tumour, Kaposi sarcoma, and rhabdomyosarcoma all accounted for one case each. The various histological diagnoses and their age variations are shown in the box and plot chart in Figure 5.

A total of 35 cases (50%) were malignant or preinvasive malignancy. Twenty-five cases (83.3%) of the 30 cases of OSSN are positive for HIV. Patients with carcinoma in situ were found to be positive for HIV in 8 cases (61.5%). All the cases of squamous cell carcinoma were positive for HIV. The only case of ocular Kaposi sarcoma found in the lid conjunctiva of a 30-year-old man was positive for HIV. None of the cases of squamous papilloma were positive for HIV. The only case of adenoid cystic carcinoma which was found on the left eyelid of a 38-year-old man was negative for HIV. Chi square test (likelihood ratio) of association between HIV status and histologic diagnosis showed a significant relationship, *χ* ^2^ (*N* = 66) = 121.748, *p* < 0.001. Twenty-six (100%) of the HIV positive cases had malignant ocular lesions. Only four (14.3%) of the HIV negative cases had malignant ocular lesions. Pearson's chi square showed a significant association between HIV status and malignancy, *χ* ^2^ (*N* = 66) = 92.55, *p* < 0.001.

Figure 1 shows the two highest frequencies to be in age groups 30–39 and 20–29. Only 59 cases had specific details on the age of the patient.

Figure 2 shows the mean age of HIV negative malignant and benign cases to be similar. The mean age of HIV positive malignant cases is considerably higher than that of HIV negative cases.

Figure 3 shows a photomicrograph of an invasive squamous carcinoma of the conjunctiva in a 35-year-old HIV positive man (haematoxylin and eosin stained section ×40).

Figure 4 shows a photomicrograph of a conjunctival squamous papilloma in a 43-year-old man who was HIV negative (haematoxylin and eosin stained section ×40).

Figure 5 shows box plot of mean age and age variation of the various histological diagnoses.

## 4. Discussion

Ocular lesions constitute a major health challenge in sub-Saharan Africa. This is compounded by the fact that patients present late to the hospitals, many of which are ill equipped and poorly staffed [6].

The results show slight preponderance of ocular lesions in males. It is well known that males have a higher socioeconomic and cultural status compared to females [7]. Males hence can more easily access health institutions. Males tend to have better care. This may explain the difference in the overall percentages. There was no significant difference in the percentages of sex of patients with OSSN. This is in conformity with reports from sub-Saharan Africa but differs from that of most parts of the globe where there is substantial difference between the two sexes. Some authors had suggested hormonal dysfunction and androgen receptor expression by some of these OSSN [8–11]. This probably plays little or no role in the cases seen in our study.

The most common malignant lesion was squamous cell carcinoma. This is similar to reports from Nigeria and other parts of the globe [1–3, 12, 13]. Retinoblastoma was rare and found only in children. Retinoblastoma is known to be the most common childhood malignancy in this region [1, 3, 14]. Our finding in this regard conforms to other reports.

The mean age of patients with carcinoma in situ was 1.3 years lower than that for patients with squamous cell carcinoma. Although this is not a direct measurement of disease progression, this finding seems to suggest that if left untreated patients will probably develop invasive carcinoma within the first two years of being diagnosed with in situ carcinoma. This may also be applicable to cases with microinvasive carcinoma which was not detected.

There is little or no difference in the mean age of both benign and malignant lesions of HIV negative cases. This is not unexpected as the benign lesions identified are not preinvasive or directly related to the malignant tumours identified and are probably independent. There is however a markedly higher mean age of malignant HIV positive cases when compared with the malignant HIV negative cases. The weighted average of the malignant ocular childhood tumours which were found to be HIV negative contributes to the disparity.

In recent times, Ocular Surface Squamous Neoplasia (OSSN), with a spectrum extending from mild dysplasia to invasive squamous carcinoma, had become a major health challenge all over the world [15–17]. Although the prevalence of OSSN in HIV positive patients is relatively low, many of the cases of OSSN are positive for HIV and this may be the first clinically identified lesion in such patients [15–18]. This finding had been strongly associated with the emergence of the HIV pandemic which has impacted more the health care delivery system especially in sub-Saharan Africa [19–21]. The strength of the association between OSSN and HIV infection varies from one locality to another. Exposure to ultraviolet radiation and high risk serotypes of human papilloma virus have also been strongly associated with the occurrence of OSSN [22]. Our study shows a strong association between ocular squamous cell carcinoma and HIV infection. HIV is well known to cause immunosuppression which increases the risk of malignant tumours especially malignancies related to oncogenic DNA viruses. It therefore becomes imperative that HIV screening should be included as part of the routine workup of patients diagnosed with invasive squamous cell carcinoma (as well as those diagnosed with carcinoma in situ) of the orbitoocular region [23].

It is not surprising that none of the patients with squamous papilloma was HIV positive. Squamous papilloma is widely known to be one of the most common benign ocular surface epithelial tumours. It has not been associated with the high risk HPV serotypes or immunosuppression.

Pterygium had been identified as the most common nonneoplastic lesion excised for histopathology [24]. A probably spurious finding in this study is the relatively low number of cases of pterygium. This does not conform to findings in quite a number of centres. This may be due to the fact that the study centre is a referral centre and usually deals with the most sophisticated cases. It may also be due to the fact that majority of cases that are excised based on a clinical suspicion of being pterygium are not routinely sent for histopathology. It is necessary to further investigate this and encourage ophthalmologists to always submit all excisions for laboratory confirmation if found to be true.

## 5. Conclusion

There was no significant difference in the sex distribution of patients with OSSN. It is probable that if left untreated patients diagnosed with OSSN will develop squamous cell carcinoma within 2 years of the initial diagnosis. HIV infection probably plays a significant role in the pathogenesis of ocular squamous cell carcinoma.
